# Rapid COVID-19 Antigen Testing in Croatia: Risk Perception Plays an Important Role in the Epidemic Control

**DOI:** 10.3389/fpubh.2021.708907

**Published:** 2021-07-27

**Authors:** Dragan Primorac, Vitorio Perić, Vid Matišić, Vilim Molnar, Renata Zadro, Adriana Vince, Gordan Lauc, Ozren Polašek

**Affiliations:** ^1^St. Catherine Specialty Hospital, Zagreb, Croatia; ^2^School of Medicine, JJ Strossmayer University of Osijek, Osijek, Croatia; ^3^Department of Pediatrics, University of Split School of Medicine, Split, Croatia; ^4^Eberly College of Science, The Pennsylvania State University, University Park, State College, PA, United States; ^5^The Henry C. Lee College of Criminal Justice and Forensic Sciences, University of New Haven, West Haven, CT, United States; ^6^Faculty of Dental Medicine and Health, School of Medicine, University “Josip Juraj Strossmayer, Osijek, Croatia; ^7^Medical School, University of Rijeka, Rijeka, Croatia; ^8^Medical School REGIOMED, Coburg, Germany; ^9^Medical School, University of Mostar, Mostar, Bosnia and Herzegovina; ^10^University Hospital for Infectious Diseases Dr. Fran Mihaljević, Zagreb, Croatia; ^11^School of Medicine, University of Zagreb, Zagreb, Croatia; ^12^Genos Ltd., DNA Laboratory, Zagreb, Croatia; ^13^Faculty of Pharmacy and Biochemistry, The University of Zagreb, Zagreb, Croatia; ^14^Department of Public Health, University of Split School of Medicine, Split, Croatia

**Keywords:** SARS-CoV-2, risk, perception, antigen, diagnostic, pooling

## Abstract

**Aim:** To explore the clinical presentation and epidemiological history of the subjects who underwent SARS-CoV-2 antigen testing.

**Methods:** We included 1,000 consecutive subjects who presented themselves at the diagnostic clinic in Croatia and analyzed their symptoms and epidemiological history. All subjects were classified into three groups, according to their reason of arrival; symptomatic, contacts of confirmed patients, and those who were tested due to administrative reasons.

**Results:** On average, there were 24% of positive antigen results; the positivity rate was 51% among symptomatic, 16% in contacts, and 5% of administrative patients. The commonest symptoms of the disease included febrility and anosmia. We developed a clinical score to predict SARS-CoV-2 positivity, which had an area under the curve of 79.3 [95% confidence intervals (CI) 75.8–82.8]. Contact with the isolated person [odds ratio 0.54 (95% CI 0.31–0.94)] and international travel had a protective effect [0.20 (0.09–0.43)], suggesting that risk perception and mandatory pretravel measures had a key role in the determination of the infection risk.

**Conclusions:** A combination of clinical symptoms can have reasonable predictive power for an antigen-positive test result. Risk perception seems to have a role in the epidemic spread, probably *via* stricter adherence to personal preventative measures.

## Introduction

The SARS-CoV-2 was discovered due to Wuhan viral pneumonia cases in 2019 and a pandemic was declared by the WHO on March 11, 2020. Common clinical signs of a person infected with SARS-CoV-2 include respiratory symptoms, fever, cough, shortness of breath, loss of smell and taste, and dyspnea (1). In more severe cases, the infection can cause bilateral pneumonia, severe acute respiratory syndrome, kidney failure, and even death (2). The SARS-CoV-2 virus is mainly transmitted *via* droplets generated by sneezing, coughing, and talking, as well as through contaminated hands since the virus can survive on various surfaces (3, 4). The diagnosis of COVID-19 encompasses clinical criteria, epidemiological history, and molecular detection of viral RNA in clinical samples. However, due to the growing number of people infected and tested positive on SARS-CoV-2 worldwide, it became necessary to develop easier, cheaper, and more available testing methods that could answer the need for increased testing. One of such methods is the rapid antigen test based on the direct detection of SARS-CoV-2 viral proteins in nasopharyngeal or nasal swabs using lateral flow immunoassay (5–7). The specificity of the antigen test is similar to the specificity of RT-PCR and ranges from 93.9 to 100%, while sensitivity varies from 84.0 to 97.6% compared to RT-PCR (8, 9). Rapid tests are very informative for persons who have been in contact with confirmed patients with COVID-19 and are particularly useful for testing in higher-risk settings (nursing homes and student dormitories) when repetitive testing can easily detect persons with SARS-CoV-2 infection and prevent the spread of the infection. Even though rapid antigen tests are less sensitive than nucleic acid amplification tests, they provide results in approximately 15 min and present an excellent alternative and addition to the PCR testing (10).

This study aimed to analyze the association of symptoms and epidemiological history with the results of antigen testing and to explore the extent of clinical symptoms related to SARS-CoV-2 infection.

## Materials and Methods

### Study Design

This was an observational study, based on the results of the COVID-19 antigen tests. The sample consisted of 1,000 consecutive subjects who presented themselves at the diagnostic clinic of the St. Catherine Specialty Hospital in Zagreb, Croatia. All the subjects were classified in one of three groups; symptomatic (who presented themselves with any combination of symptoms that might be associated with the COVID-19 disease), contacts of confirmed patients, and administrative reasons (which most commonly included a mandatory preoperative assessment). The subjects were recruited from October 2020–January 2021. All of the participants signed informed consent to participate in the study. The data for the study were based on unlinked testing results without any identifiable information, not requiring additional ethical approval.

### Measurements

To collect a nasopharyngeal swab sample, a sterile swab was inserted into the nostril of the patient, gently pushing the swab into the posterior nasopharynx and rotating against the nasal wall. After the sample collection, we used two rapid antigen tests with similar declared specificity and sensitivity (SARS-CoV-2 Rapid Antigen Test, SD Biosensor, the Republic of Korea distributed by Roche Diagnostics GmbH, Germany and COVID-19 Ag Test, von Minden GmbH, Germany). All tests were applied in accordance with the instructions of the manufacturer. The Ethical approval for the study was issued by the Ethical board of the Sv. Katarina hospital.

### Statistical Methods

The numerical data were analyzed with *t*-test, while the categorical were analyzed with chi-square test. We also created a logistic regression model, which incorporated multiple predictor variables to analyze the symptoms or the leading epidemiological risks. We also developed a ROC curve, by using the lead results of the logistic regression model, aiming to assess the clinical symptoms in the prediction of antigen test positivity. All the analyses were performed in R, with significance set at *P* < 0.05.

## Results

We included 1,000 consecutive subjects in this study, who have presented themselves at the diagnostic clinic. Among them, there were 337 symptomatic (33.7%), 291 were contacts (29.1%), while 372 (37.2%) were tested for administrative reasons, most commonly due to preoperative protocol that included mandatory testing (37.2%). Overall, the positivity rate was 23.6% on all tests. The breakdown according to the reason of testing yielded 50.7% positive antigen tests among the symptomatic subjects, 16.2% among contacts of people infected with SARS-CoV-2, and 4.8% of positive results in the group of subjects tested for administrative reasons. The three groups did not differ according to age, but subjects who were diagnosed for administrative reasons were insignificantly older (Table 1).

**Table 1:** Sample breakdown according to the group, age, and antigen testing results.

	**Symptomatic (*n* = 337)**	**Contacts (*n* = 291)**	**Administrative (*n* = 372)**	***P***
Age, years; mean ± SD [min–max]	38.3 ± 14.6 [1–79]	38.1 ± 12.8 [3–78]	40.4 ± 13.2 [5–83]	0.059
Antigen-positive; *n* (%)	171 (50.7)	47 (16.2)	18 (4.8)	<0.001

Positively tested subjects reported an interesting blend of symptoms, with the greatest odds ratios for anosmia, fever, muscle pain, and cough. Interestingly, diarrhea seemed to be inversely associated with the positive result on the SARS-CoV-2 antigen test (Table 2). We then entered these four predictor variables in the score development, alongside age, which was also significant in the logistic regression model. The final model was created as the sum of binary results for the three significant symptoms, minus diarrhea, times age, or COVID score = (fever + cough + anosmia-diarrhoea) ^*^ age. The use of ROC indicated that the score had good predictive power, yielding an area under the curve of 79.3 [95% CI 75.8–82.8].

**Table 2:** Reported symptoms in subjects with positive antigen test results, logistic regression.

	***P***	**OR [95% CI]**
Age	0.033	1.01 [1.00–1.03]
Fever	** <0.001**	**5.94 [4.07–8.66]**
Sore throat	0.209	1.42 [0.82–2.43]
Loss of taste	0.239	0.56 [0.22–1.47]
Anosmia	** <0.001**	**8.56 [3.86-18.99]**
Lack of energy	0.840	1.07 [0.54–2.13]
Fatigue	0.373	0.71 [0.33–1.51]
Cough	**0.009**	**1.88 [1.17**–**3.03]**
Runny nose	0.858	1.06 [0.54–2.10]
Dyspnoea	0.054	2.65 [0.98–7.16]
Nausea	0.111	0.25 [0.05–1.37]
Diarrhoea	**0.005**	**0.11 [0.02**–**0.51]**
Vomit	0.294	0.28 [0.03–2.97]
Muscle pain	**0.007**	**2.31 [1.26–4.26]**
Headache	0.039	1.65 [1.03–2.65]
Back pain	0.511	1.46 [0.47–4.47]
Chest pain	0.337	0.49 [0.12–2.10]

*Significant values are provided in bold*.

The final analytic step was aimed at uncovering the epidemiological risks associated with the antigen-positive result. The first model that was used suggested that self-isolation within the past 2 weeks had the greatest odds of a positive result. In the subsequent model, we had excluded subjects who reported this (as they might have required a retesting after the self-isolation period had ceased). The reduced model suggested two significant results, contact with the isolated person within the past 2 weeks and international travel within the past 2 weeks; both models had a good fit, demonstrating a protective effect (Table 3).

**Table 3:** Exposures and risks for antigen positive test results, logistic regression.

**Predictor**	**Full model**		**Excluded subjects who were ordered to self-isolate within the past 14 days**	
	***P***	**OR [95% CI]**	***P***	**OR [95% CI]**
Age	0.095	1.01 [1–1.02]	0.535	1.01 [0.99–1.019]
Ever been in self-isolation	0.016	0.55 [0.34–0.9]	0.398	0.7 [0.3–1.611]
Experienced self-isolation within past two weeks	<0.001	2.87 [2.05–4.02]		
Live with a person who was positive	0.716	1.09 [0.69–1.73]	0.240	1.43 [0.79–2.584]
Contact with positive person within past two weeks	0.740	0.93 [0.59–1.46]	0.948	1.02 [0.58–1.803]
Contact with isolated person within past 2 weeks	0.030	0.54 [0.31–0.94]	0.036	0.37 [0.15–0.937]
Participated in an event with over 15 people within past two weeks	0.173	1.53 [0.83–2.8]	0.134	1.74 [0.84–3.609]
International travel within past two weeks	<0.001	0.20 [0.09–0.43]	<0.001	0.21 [0.09–0.501]
*Hosmer-Lemeshow test*	0.561		0.603	

## Discussion

The results of this study demonstrate that the combination of clinical symptoms can have a reasonable predictive power for an antigen-positive test result. In addition, these results show a substantial discrepancy of result outcomes in three groups of subjects. This finding is very interesting for increasing the effectiveness of the diagnostic process. The information on the *a-priori* chance for a positive result and population prevalence are critical for pooling, which aims to optimize the diagnostic procedures by reducing the cost of diagnostics. One of the most interesting results of this study is, therefore, the need for inclusion of information on the reason for testing to the actual testing process, knowing that certain pooling methods have the best savings in situations of low population prevalence, while their savings decrease as the population prevalence of the disease rises.

These results also show that risk perception plays a moderate but significant role in the antigen-confirmed disease status. This is to a degree moderated by the requirement for international travel (11–13), which is very strict and may elicit the need for multiple tests and strict adherence to personal protective equipment and epidemiological measures. Regardless of the modes of use, antigen testing is now established as a useful tool in diagnostic capabilities (14–16).

Clinical presentation of COVID-19-positive subjects was in line with previous studies, with cough, fever, and loss of smell being the most commonly reported symptoms (2). Interestingly, we have shown that diarrhea was inversely correlated with disease risk, which was in disagreement with a recent systematic review that had suggested that gastrointestinal symptoms were experienced by 13% of all patients (17). Although the ROC estimates in this study seem rather high, new approaches predicting critical patients have managed to yield even greater estimates (18).

The results of this study must be considered within the wider-sense societal epidemiological situation, marked by the strict initial quarantine and favorable population response in the early epidemic stages (19, 20). It could be argued that the epidemic dynamics may affect the situation, therefore, the results obtained here might not be relevant for all time points of the epidemic; this was already identified in the past, suggesting the need for constant monitoring and provision of timely-sensitive data for decision making (20). Additionally, the sample composition is also a possible source of bias, including the patients who presented themselves in the private clinic, preventing generalizability to the entire population. Nevertheless, the results do show that the diagnostic process should include clinical information, and not be completely unlinked. This may increase the effectiveness of the diagnostic process, by the ability to employ pooling in favorable instances (21). A better understanding of the risk determinants and improvements of the diagnostic process are the key components of reactive epidemic management.

## Data Availability Statement

The raw data supporting the conclusions of this article will be made available by the authors, without undue reservation.

## Ethics Statement

The studies involving human participants were reviewed and approved by Ethical board of the Sv. Katarina Hospital. The patients/participants provided their written informed consent to participate in this study.

## Author Contributions

VP, VMa, VMo, RZ, GL, and DP conceived the study and designed the study in collaboration. OP analyzed the data. VP, VMa, VMo, AV, and RZ wrote the first draft of the manuscript. OP and DP critically revised the manuscript. All the authors interpreted the data and contributed to subsequent drafts of the manuscript, and all the authors have seen and approved the final version.

## Conflict of Interest

DP, GL, and OP are scientific advisors to the Government of the Republic of Croatia for COVID-19 response, which had no influence in the decision to prepare the manuscript, its preparation or the selection of the journal where the manuscript would be submitted. The remaining authors declare that the research was conducted in the absence of any commercial or financial relationships that could be construed as a potential conflict of interest.

## Publisher's Note

All claims expressed in this article are solely those of the authors and do not necessarily represent those of their affiliated organizations, or those of the publisher, the editors and the reviewers. Any product that may be evaluated in this article, or claim that may be made by its manufacturer, is not guaranteed or endorsed by the publisher.
